# Charting the Unknown: Green Urine After Propofol in Pediatric Trauma

**DOI:** 10.7759/cureus.56588

**Published:** 2024-03-20

**Authors:** Reda El Farh, Othman Moueqqit, Zakaria Bouayed, Ilias El Kadiri Boutchich, Omar Alaoui Mhammedi, Wael El Fergui, Ghizlane El Aidouni, Houssam Bkiyar, Brahim Housni

**Affiliations:** 1 Faculty of Medicine, Mohammed First University, Oujda, MAR; 2 Anesthesiology and Intensive Care Unit Department, Mohammed VI University Hospital, Oujda, MAR; 3 General Medicine, Faculty of Medicine and Pharmacy, Mohammed First University, Oujda, MAR

**Keywords:** critical care management, chromatic alterations, pediatric head trauma, propofol, green urine

## Abstract

The phenomenon of green urine discoloration, while rare, represents a captivating clinical puzzle that challenges the distinction between benign and pathological conditions. In this report, we present an intriguing case involving a 15-year-old trauma patient admitted following a motorcycle collision, where the ensuing unconsciousness necessitated propofol induction for intubation and sedation. Remarkably, around 48 hours post-admission, the patient displayed green urine discoloration, which resolved spontaneously within just 12 hours. This case serves as a compelling illustration of the uncommon occurrence of propofol-induced green urine in the context of critical care management, underscoring the imperative need to discern and appreciate medication-related chromatic alterations in urine.

## Introduction

Green urine discoloration can occur due to various factors, such as medications, dyes, infections, ingested substances, and numerous other etiological agents [1]. Propofol (2,6 diisopropylphenol), a frequently employed anesthetic agent, has, in infrequent instances, been documented as a potential cause of green urine discoloration [1]. This can lead to undue concern for healthcare providers and the necessity for additional laboratory testing when not recognized by the attending clinician [2]. The scientific literature contains limited documentation concerning the phenomenon of urine turning green as a consequence of propofol administration [2]. In this case study, we present an instance of green urine discoloration observed in a pediatric patient who received propofol sedation following a head trauma.

## Case presentation

A 15-year-old patient with no notable medical history was admitted to the hospital for severe trauma following a motorcycle collision with a car. The initial clinical examination during the resuscitation process revealed an unconscious patient with a Glasgow Coma Scale score of 11/15, exhibiting agitation. The patient had equal and reactive pupils, with a blood pressure of 110/50 mmHg, heart rate of 95 beats per minute, respiratory rate of 16 cycles per minute, and oxygen saturation of 97% on room air.

A primary survey revealed a lip laceration and multiple abrasions on the face and both knees. Rapid sequence induction was performed, involving the administration of 100 mg of propofol, 70 mg of rocuronium, and 200 μg of fentanyl. The patient was intubated using a size 4 laryngoscope blade, and an endotracheal tube of size 6.5 was inserted and secured at a depth of 21 cm. Controlled ventilation was initiated with the following parameters: a tidal volume of 420 mL, respiratory rate of 14 breaths per minute, positive end-expiratory pressure set at 3 cmH_2_O, fraction of inspired oxygen of 100%, and an inspiratory to expiratory ratio of 1:2.

**Figure 1:**
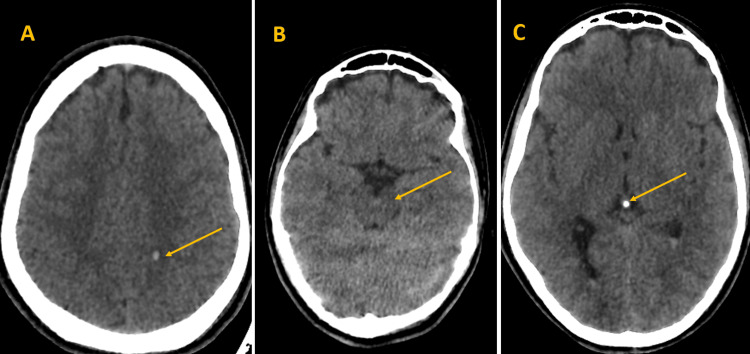
Cerebral region imaging showing a minor hemorrhagic contusion in the left parietal lobe (A), midbrain hemorrhage (B), and intraventricular hemorrhage (C).

**Figure 2:**
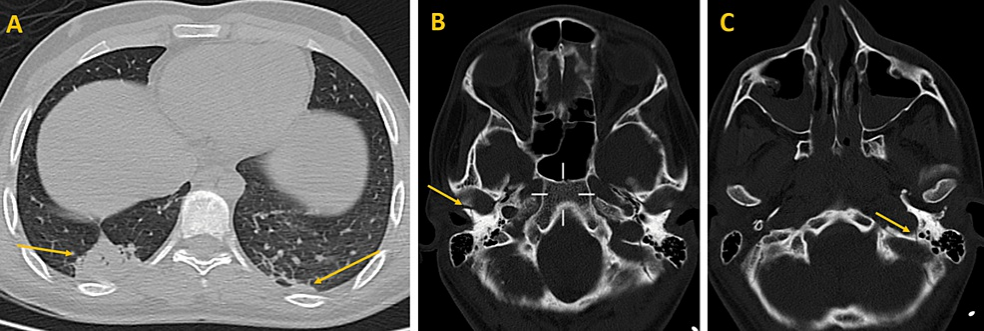
Thoracic and osseous region findings. A: Thoracic imaging indicating parenchymal consolidations in the lower lobes. B: Osseous window imaging highlighting a fracture line in the nasal bones along with a fracture line extending from the right condyle of the mandible to the ipsilateral tympanic bone. C: Osseous window imaging showing a fracture line in the left jugular foramen.

Nasogastric and urinary catheterizations were performed, resulting in 200 mL of normal-looking urine being drained by the urinary catheter. Subsequently, a whole-body CT scan was conducted, revealing multiple findings. In the cerebral region, a minor left parietal hemorrhagic contusion was noted, along with a hemorrhage in the midbrain. Additionally, an intraventricular hemorrhage was observed (Figure 1). In the thoracic region, bilateral lower lobe parenchymal consolidations were noted, suggestive of contusions (Figure 2). Furthermore, in the osseous window, fractures were identified, including a fracture line of the nasal bones, a fracture line extending from the right condyle of the mandible to the ipsilateral tympanic bone, and a fracture line of the left jugular foramen (Figure 2).

Laboratory tests, including renal and hepatic function, showed normal results. Arterial blood gas analysis revealed normal values, with lactate levels measured at 0.68 mmol/L.

**Figure 3:**
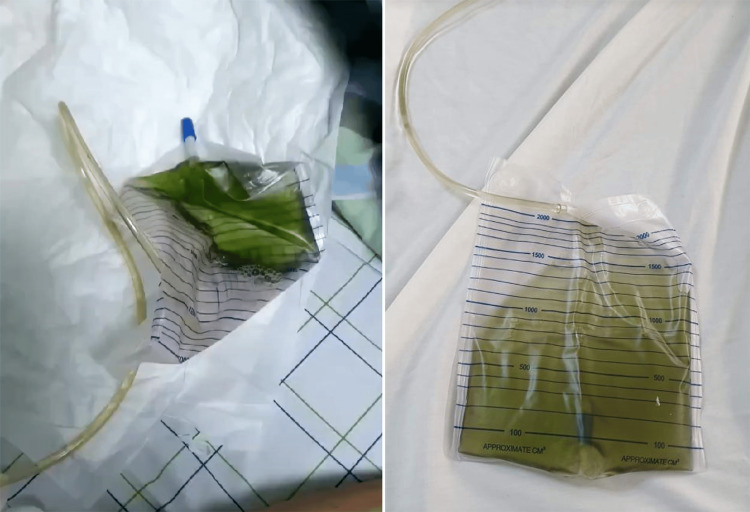
Grass-green-colored urine with Foley catheter in situ.

A consultation with the neurosurgery team was obtained, and the decision was made to maintain sedation using propofol and midazolam, as there was no indication for surgical intervention. Additionally, the patient was started on levetiracetam 500 mg every 12 hours for seizure prophylaxis. Approximately 48 hours later, a change in urine color to green was noted (Figure 3). However, subsequent laboratory investigations, including urine culture, remained unremarkable.

Approximately 12 hours later, the green discoloration of the urine spontaneously resolved. After 14 days, the patient was successfully extubated and subsequently transferred to the neurosurgery department for further care.

## Discussion

Numerous intrinsic and extrinsic elements have the potential to induce a green hue in urine. Medications such as cimetidine, promethazine, amitriptyline, indomethacin, metoclopramide, methocarbamol, and flutamide, along with dyes such as methylene blue, indigo blue, biliverdin, and food dyes, as well as metabolic conditions such as Hartnup disease, *Pseudomonas aeruginosa* infections, and vesical fistulas, constitute additional plausible etiological factors for the manifestation of green urine discoloration [2].

Pedersen et al. [3] documented that the development of discoloration becomes evident when the clearance of propofol surpasses hepatic elimination, subsequently leading to extrahepatic elimination of propofol. Whereas Shioya et al. [4] noted that green urine was associated with enterohepatic circulatory issues due to constipation and peristalsis impairment, as well as enhanced carrier protein utilization from albumin and erythrocyte administration, along with extrahepatic glucuronidation in the kidneys. However, our patient had no prior or current hepatic complications according to the biological evaluations. Hence, while phenolic derivatives of propofol are suspected culprits for the green urine discoloration, its exact origin remains unclear.

The green discoloration resolved on its own within about 12 hours after propofol discontinuation, consistent with previous reports that have noted variations in onset times, ranging from as early as two hours to up to two days after discontinuing propofol [5].

This case highlights the need for a comprehensive medical history and a thorough evaluation of medication usage, as even commonly administered drugs such as propofol can have unexpected effects. It serves as a reminder for healthcare professionals to maintain vigilance in identifying such atypical presentations and encourages further exploration of the underlying mechanisms behind this phenomenon.

## Conclusions

This case of green urine following propofol in a pediatric trauma patient, though rare and initially puzzling, ultimately resolved spontaneously without consequence. It underscores the need for careful monitoring, recognition, and management of medication-related urine color changes. This contributes to medical knowledge, emphasizing the importance of sharing such cases within the healthcare community, demonstrating that even uncommon occurrences can be effectively managed with clinical expertise.
